# Preliminary Feasibility of a Novel Mind-Body Program to Prevent Persistent Concussion Symptoms Among Young Adults With Anxiety: Nonrandomized Open Pilot Study

**DOI:** 10.2196/64540

**Published:** 2024-11-08

**Authors:** Molly Elizabeth Becker, Nadine Stratton Levey, Gloria Y Yeh, Joseph Giacino, Grant Iverson, Noah Silverberg, Robert A Parker, Ellen McKinnon, Caitlin Siravo, Priyanca Shah, Ana-Maria Vranceanu, Jonathan Greenberg

**Affiliations:** 1 Psychology Department Suffolk University Boston, MA United States; 2 Center for Health Outcomes and Interdisciplinary Research Massachusetts General Hospital Boston, MA United States; 3 Harvard Medical School Boston, MA United States; 4 Department of Medicine Beth Israel Deaconess Medical Center Boston, MA United States; 5 Spaulding Rehabilitation Hospital Charleston, MA United States; 6 Department of Psychiatry Massachusetts General Hospital Boston, MA United States; 7 Department of Physical Medicine and Rehabilitation Spaulding Rehabilitation Hospital and Spaulding Research Institute Harvard Medical School Charlestown, MA United States; 8 Mass General Hospital for Children Sports Concussion Program Boston, MA United States; 9 Home Base A Red Sox Foundation and Massachusetts General Hospital Program Charlestown, MA United States; 10 Department of Psychology University of British Columbia Vancouver, BC Canada; 11 Rehabilitation Research Program Vancouver Coastal Health Research Institute Vancouver, BC Canada; 12 Biostatistics Center Massachusetts General Hospital Boston, MA United States; 13 Dr Robert C Cantu Concussion Center Emerson Hospital Concord, MA United States

**Keywords:** concussions, mind-body, preventions, young adults, feasibility, persistence, open pilot, mind-body program, preliminary feasibility, mild traumatic brain injuries, United States, adults, psychological factors, mind-body interventions

## Abstract

**Background:**

Concussions are common, particularly among young adults, and often are associated with persistent, debilitating, and hard-to-treat symptoms. Anxiety and concussion symptoms often amplify each other, and growing evidence indicates that anxiety plays a key role in symptoms persistence after concussion. Targeting anxiety early after concussion may be a promising means of helping prevent persistent concussion symptoms in this population. We developed the Toolkit for Optimal Recovery after Concussion (TOR-C), the first mind-body program tailored for young adults with a recent concussion and anxiety, aiming to prevent persistent concussion symptoms.

**Objective:**

This study aims to conduct an open pilot of TOR-C to test preliminary feasibility, signal of change in measures, and treatment perceptions.

**Methods:**

Five young adults (aged 18-24 years) attended 4 weekly one-on-one live video sessions with a clinician. Participants completed questionnaires measuring treatment targets (ie, pain catastrophizing, mindfulness, fear avoidance, limiting behaviors, and all-or-nothing behaviors) and outcomes (ie, postconcussive symptoms, physical function, anxiety, depression, and pain) at baseline, immediately following the intervention, and 3 months after intervention completion. At the conclusion of the program, participants attended a qualitative interview and provided feedback about the program to help optimize study content and procedures.

**Results:**

Feasibility markers were excellent for credibility and expectancy (5/5, 100% of participants scored above the credibility and expectancy scale midpoint), client satisfaction (4/5, 80% of participants scored above the Client Satisfaction Questionnaire midpoint), therapist adherence (97% adherence), acceptability of treatment (5/5, 100% of participants attended 3 or more sessions), adherence to homework (87% home practice completion), and feasibility of assessments (no measures fully missing). The feasibility of recruitment was good (5/7, 71% of eligible participants agreed to participate). There were preliminary signals of improvements from pre-post comparisons in treatment targets *(d*=0*.*72-2.20) and outcomes *(d*=0.41-1.38), which were sustained after 3 months (*d*=0.38-2.74 and *d*=0.71-1.63 respectively). Exit interviews indicated overall positive perceptions of skills and highlighted barriers (eg, busyness) and facilitators (eg, accountability) to engagement.

**Conclusions:**

TOR-C shows preliminary feasibility, is associated with a signal of improvement in treatment targets and outcomes, and has the potential to support recovery from concussion. The quantitative findings along with the qualitative feedback obtained from the exit interviews will help optimize TOR-C in preparation for an upcoming randomized controlled trial of TOR-C versus an active control condition of health education for concussion recovery.

**International Registered Report Identifier (IRRID):**

RR2-10.2196/25746

## Introduction

Concussions or mild traumatic brain injuries impact approximately 30% of adults in the United States [1]. The impact of concussions can be broad and debilitating, with a range of associated cognitive (eg, attention and memory deficits, brain fog), emotional (eg, anxiety, emotion dysregulation, irritation), and physiological (eg, photosensitivity, headaches, nausea) symptoms, which affect many life domains. Persistent symptoms after a concussion are common (64% by some estimates) [2] and difficult to treat. Developing interventions to help prevent persistent concussion symptoms is a high priority.

Psychological factors play a key role in recovery from concussion [3-8]. Anxiety is associated with the severity of concussion symptoms and is one of the strongest predictors of recovery trajectory and transition from acute to persistent concussion symptoms [7-9]. This may be in part because anxiety symptoms and common symptoms following concussion overlap (eg, headache, nausea, fatigue, irritability, insomnia), and they mutually amplify. Anxiety is highly prevalent in young adults and rapidly increasing [10], placing this age group at risk for developing persistent symptoms. Targeting anxiety early on after a concussion among young adults may be a promising yet unexplored means of helping to prevent persistent concussion symptoms.

Mind-body interventions are effective for treating anxiety [11-13] and can benefit individuals after concussion [14,15]. Nevertheless, to date, there are no mind-body interventions developed specifically for young adults with a recent concussion and anxiety, who are at risk for symptom persistence and unique challenges [5] with unique treatment needs and preferences [16].

**Figure 1:**
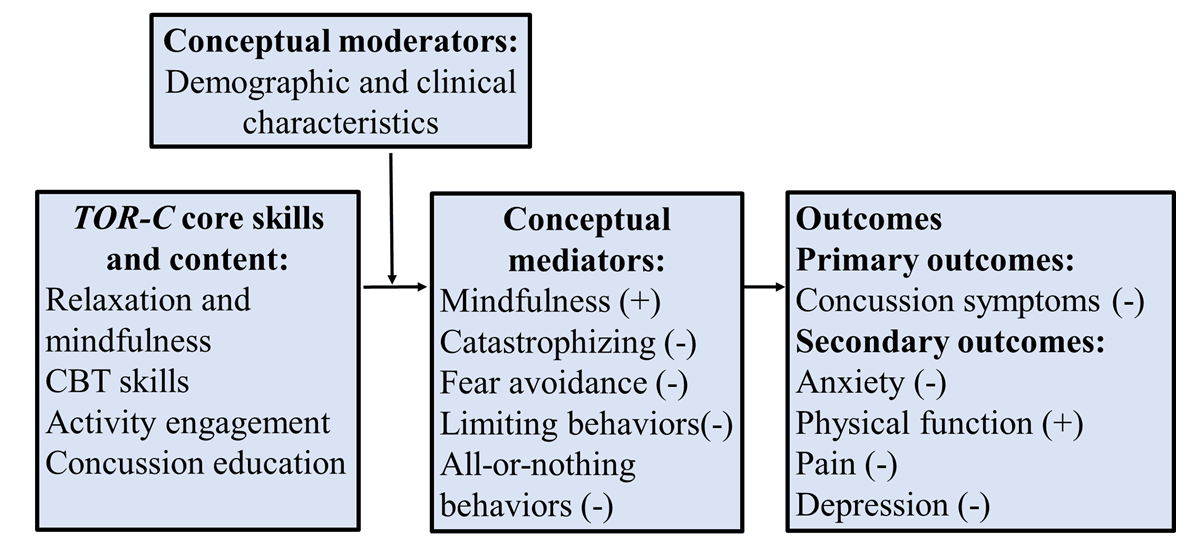
Conceptual model of TOR-C that outlines psychosocial processes impacting anxiety and concussion symptoms. CBT: cognitive behavioral therapy; TOR-C: Toolkit for Optimal Recovery after Concussion.

To address this gap, we developed the Toolkit for Optimal Recovery after Concussion (TOR-C), a 4-session mind-body program delivered over live video aiming to prevent persistent concussion symptoms in young adults with co-occurring anxiety. Adapted from Toolkit for Optimal Recovery after Orthopedic Injury for chronic pain after orthopedic injuries [17], the program teaches mind-body skills (eg, body scan, deep breathing, and mindfulness), cognitive-behavioral skills (eg, reframing), acceptance and commitment skills (eg, acceptance and engagement with valued activities), and psychoeducation about concussion recovery. Figure 1 depicts the conceptual model of TOR-C. The program is informed by the fear avoidance theoretical model, originally developed to explain the development of chronic pain [18,19] but receiving growing empirical support for concussions [20-22]. The model depicts the interaction of psychosocial processes (eg, anxiety, symptom catastrophizing, and activity avoidance), which can result in symptom persistence. The TOR-C skills directly target these processes, with the intention of preventing concussion symptoms from becoming persistent.

The development of TOR-C followed the National Center for Complementary and Integrative Health (NCCIH) framework for developing and testing mind-body interventions [23]. This framework encourages researchers to define treatment targets, measure preliminary changes in treatment outcomes, establish feasibility, and refine interventions prior to efficacy trials. Accordingly, we first conducted qualitative live videos with young adults who were recently concussed and experienced co-occurring anxiety to understand their experiences after their concussion, gauge their treatment needs, and document their perceptions of intervention components [5,16,24]. We used these data to develop the TOR-C intervention manual and treatment procedures.

The purpose of this open pilot study is to test preliminary feasibility markers [4] of TOR-C and signals of improvement in intervention targets (eg, pain catastrophizing, fear avoidance, limiting behaviors, all-or-nothing behaviors, and mindfulness) and treatment outcomes (eg, concussion symptoms, anxiety, depression, pain, and physical function). Further, we aimed to gather feedback about the intervention, using qualitative exit interview interviews to refine the intervention and treatment procedures in preparation for a pilot randomized controlled trial (RCT).

## Methods

### Participants

We recruited 5 participants through provider referrals from the Massachusetts General Hospital emergency department and concussion clinics in the greater Boston area. Participants were also recruited from research invitations via Mass General Brigham’s Patient Gateway (as identified through Research Patient Database Registry queries), Partners Rally, and institutional review board–approved flyers. A trained research assistant conducted all eligibility screenings over the phone, which included assessments of suicidality and nonsuicidal self-injury. Those endorsing suicidal ideation underwent a risk assessment, including establishing a safety plan as needed. All participants reported receiving a concussion diagnosis, and this was confirmed for those referred by providers.

Inclusion criteria were (1) sustaining a concussion within 3-10 weeks of screening; (2) scoring 5 or more on the Generalized Anxiety Disorder-7 (GAD-7) scale (ie, at least mild anxiety); (3) aged 18-24 years; (4) having English fluency and literacy; (5) ability and willingness to participate in program sessions and complete questionnaires; and (6) being cleared for participation by referring provider or study staff.

Exclusion criteria were (1) participation in mind-body or cognitive behavioral therapy treatment in the past 3 months; (2) concussion in the past 2 years that resulted in symptoms lasting 3 months or more; (3) previous complicated or moderate or severe traumatic brain injuries; (4) practice of mindfulness techniques greater than 45 min/wk in the past 3 months; (5) changes to psychotropic medication in the past 3 months; (6) serious uncontrolled psychiatric comorbidity (eg, schizophrenia) or active suicidal ideation; (7) pregnancy; (8) access to secondary gains that may bias motivation (eg, pending disability claim); and (9) serious comorbidity expected to worsen in the next 6 months (eg, cancer).

### Procedures

Following phone screening, eligible and interested participants received a digital consent form to review either independently or with guidance from the study research assistant (NSL). Prior to signing consent, the research assistant provided clarification and information for any participants with questions or concerns pertaining to the study. Following consent, the research assistant scheduled the participants for their 4 TOR-C program sessions and provided instructions for downloading and using Zoom (Zoom Video Communications) if necessary. Participants then received a survey link through a secure Health Insurance Portability and Accountability Act–approved platform (REDCap [Research Electronic Data Capture]; Vanderbilt University) for the quantitative baseline assessment. It was required that the surveys be completed in full prior to the first session.

Participants completed 4 weekly approximately 45-minute TOR-C sessions. All sessions were held 1:1 over Zoom and guided by a clinical psychologist (JG) with over 20 years of experience in mind-body and mindfulness-based practices. Procedures for the quantitative assessments were repeated directly following the fourth session and 3 months later. All participants completed a 30-minute exit interview where they provided feedback on the program, guided by a semistructured interview script.

### TOR-C Program

The TOR-C program was developed to meet the needs of young adults (aged 18-24 years) with recent concussion and comorbid anxiety. Adapted from the Toolkit for Optimal Recovery after Orthopedic Injury [17], the program consists of 4 program sessions and aims to teach (1) mind-body skills (eg, body scan, deep breathing, and mindfulness) to help cope with and deintensify concussion-related anxiety; (2) cognitive behavioral therapy skills (eg, reframing); (3) acceptance and commitment skills (eg, engagement with valued activities); and (4) skills for returning to activity (eg, goal setting) with the ultimate goal of preventing persistent concussion symptoms. Sessions began with a check-in for skills practice and general well-being. The program also includes psychoeducation about concussions and recovery, including “busting myths” about concussions (Table 1). Participants are encouraged to practice skills via optional daily text message reminders.

**Table 1:** Program content and skills for a mind-body concussion prevention program, Toolkit for Optimal Recovery after Concussion (TOR-C; N=5).

Session	Content and skills
1	Concussion education, debunking concussion myths, setting recovery goals, mind-body connection, deep breathing, body scan, and home practice
2	The “disability spiral” and “recovery path” in concussion; mindfulness, mindful breathing, and mindful STOP^a^.
3	Mindfulness of pain and discomfort, challenging unhelpful thoughts about concussion symptoms; and reengaging in physical, social, and cognitively engaging activities
4	Acceptance, problem-solving, TOR-C skills review, and plan for recovery

^a^STOP: Stop, Take a breath, Observe, Proceed.

### Measures

#### Feasibility Benchmarks

All feasibility, credibility, fidelity, and satisfaction benchmarks were determined a priori [25] in accordance with intervention development guidelines [26] and prior feasibility and intervention development studies [27]. Table 2 details each feasibility marker and its measurement.

**Table 2:** Feasibility markers for the open pilot trial of the Toolkit for Optimal Recovery after Concussion (N=5).

Marker	Criteria
Credibility	5 (100%) out of 5 participants scored above the scale midpoint (excellent)
Expectancy	4 (80%) out of 5 participants scored above the scale midpoint (excellent)
Client satisfaction score	5 (100%) out of 5 participants scored above the scale midpoint (excellent)
Feasibility of recruitment	5 (71%) out of 7 eligible participants agreed to participate (good)
Acceptability of treatment	5 (100%) out of 5 participants attended ≥ 3 out of 4 sessions (excellent)
Adherence to homework	Participants completed home practice 87% of the time (excellent)
Therapist adherence	97% adherence (excellent)
Feasibility of assessments	5 (100%) out of 5 participants had no measures fully missing (excellent)
Adverse events	None (excellent)

#### Quantitative Measures

We used the outcome measures below at baseline, immediately following the intervention, and 3 months after intervention completion.

#### Concussion Symptoms

The Post-Concussion Symptom Scale [28] presents 22 symptoms on a dimensional scale, often interpreted as follows: 0=none, 1-2=mild, 3-4=moderate, and 5-6=severe. Higher scores indicate greater symptom severity.

#### Physical Function

The World Health Organization Disability Assessment Scale (12-item version) 2.0 [29] measures one’s level of functioning in 6 domains (ie, cognition, mobility, self-care, getting along, life activities, and participation) across 12 items on a 0- to 4-point scale, with higher scores indicating greater disability.

#### Pain Catastrophizing

The Pain Catastrophizing Scale [30] is a 13-item measure aimed at assessing one’s tendency to catastrophize their pain on a scale of 0-4, with higher scores translating to higher pain catastrophizing.

#### Anxiety and Depression

The Hospital Anxiety and Depression Scale (HADS) [31] is a 14-item measure evaluating the severity of anxiety (HADS-Anxiety [HADS-A]) and depression. Scores range from 0 to 21, with higher scores indicating more symptoms. We also used GAD-7 [32], a 7-item measure assessing symptoms of anxiety on a scale of 0-3, with higher scores indicating more symptoms. The GAD-7 was used to determine meeting the minimum of “mild anxiety” cutoff during screening and was also administered at baseline as a supplemental measure to help inform the selection of an anxiety measure in a future efficacy trial.

#### Behavioral Response to Illness

The Behavioral Response to Illness Questionnaire [33] includes 2 subscales, “limiting behaviors” and “all-or-nothing behaviors,” that are comprised of 7 items and 6 items, respectively. The limiting behaviors subscale measures the tendency to avoid or refrain from various activities. The all-or-nothing behavior subscale measures the tendency for overexertion. Both are rated from 0 to 4 with higher scores indicating increased limiting or all-or-nothing behaviors.

#### Pain

The Numerical Rating Scale [34] evaluates pain severity on a scale of 0-10 at rest and during activity, with higher scores indicating greater pain, over the course of 4 items.

#### Fear Avoidance

The Fear Avoidance Behavior after Traumatic Brain Injury [35] is a 16-item measure aimed at assessing beliefs about how activity affects concussion symptoms and associated avoidance behaviors on a scale of 0 to 3, with higher scores indicating greater avoidance.

#### Mindfulness

The Cognitive and Affective Mindfulness Scale [36] measures one’s broad conceptualization of mindfulness with 12 items. Scores range from 12 to 48, with higher scores suggesting increased levels of mindfulness.

#### Exit Interviews

We used a semistructured interview guide to elicit detailed feedback from participants by addressing the following domains: (1) general impressions of TOR-C (eg, “What are your overall thoughts on the program?” and “What aspects of the program were most/least helpful to you?”); (2) impressions of program skills (eg, “What was your experience of mind-body skills in the program?” “What was your experience with increasing activity during the program?” and “What can we do better when providing information about concussion?”); (3) barriers and facilitators to home practice (eg, “What can we do better to help participants complete home practice?”); and (4) barriers or facilitators to overall program engagement (eg, “What helped [or hindered] you in consistently attending and participating in the weekly sessions?”).

### Analytic Plan

#### Quantitative Analysis

We used proportions and descriptive statistics to calculate preliminary a priori feasibility benchmarks (Table 2), consistent with Greenberg et al [25], Mace et al [27], and Vranceanu et al [17]. We calculated a preliminary signal of improvement in outcomes and intervention targets using 2-tailed paired *t* tests between pre- and postinterventions time points in addition to preintervention and follow-up time points. The small sample size inherently entails low statistical power. Therefore, in assessing changes, we relied on effect sizes (Cohen *d* of change scores from baseline to postintervention time points) rather than measures of statistical significance (eg, *P* values), while acknowledging their preliminary nature. This approach is designed to provide an initial and tentative indication of potential program effects to be tested in a future RCT, in line with guidelines for feasibility trials [37]. All analyses were conducted in IBM SPSS (version 27) and Microsoft Excel.

#### Qualitative Analysis

We analyzed qualitative data from exit interviews using rapid data analysis (RDA) [38,39]. RDA is a valid alternative to full thematic analysis, which bypasses more in-depth coding to meet the needs of intervention development [38,39]. It consists of extracting summaries and key points from each interview, followed by the creation of a response matrix consolidating the summaries of all individual interviews [38]. We used a hybrid inductive-deductive approach, following the structure of the interview guide while remaining open to novel domains expressed by participants that were not preconceptualized by the team. All exit interviews were audio recorded and facilitated by the principal investigator (JG). A trained research assistant closely listened to the recordings and drafted the matrix as well as insightful quotes. The qualitative data analysis team (the research assistant [NSL], principal investigator [JG], and lead author [MEB; a clinical psychology doctorate student]) then met to deliberate and conceptualize the findings.

### Ethical Considerations

All procedures and materials used in this study were approved by the Massachusetts General Hospital institutional review board (protocol# 2022P001521). Participants were provided written informed consent forms that illustrated their ability to opt out of treatment at any time as well as the risks and benefits of participating. All study data were deidentified and stored safely on Health Insurance Portability and Accountability Act–compliant, password-protected drives. Participants were paid up to US $110 for completing all study assessments and the exit interview.

## Results

### Participant Characteristics

Among 18 potentially eligible participants, 7 met eligibility criteria and 5 completed the program. All participants engaged in the study assessments and qualitative exit interviews. Participants predominantly identified as White (4/5, 80%), with 1 participant identifying as Eurasian, and most (3/5, 60%) identified as female. Additional demographic information is outlined in Table 3.

**Table 3:** Demographic data from participants in the open pilot trial of the Toolkit for Optimal Recovery after Concussion (N=5).

Characteristic			Value
**Age (years), mean (SD; range)**			21.4 (2.95; 18-24)
**Sex, n (%)**			
	Male	2 (40)	
	Female	3 (60)	
**Race or ethnicity, n (%)**			
	White (not Hispanic)	4 (80)	
	Multiracial (identified as Eurasian)	1 (20)	
**Marital status, n (%)**			
	Single	5 (100)	
**Education, n (%)**			
	Less than high school (less than 12 years)	1 (20)	
	Completed high school or GED^a^ (12 years)	1 (20)	
	Some college or associates degree (less than 16 years)	1 (20)	
	Completed 4 years of college (16 years)	2 (40)	
**Time from injury (days), mean (SD; range)**			41.2 (20.46; 22-76)

^a^GED: General Educational Development.

### Feasibility Markers

Table 2 details feasibility benchmarks. Credibility, expectancy, client satisfaction score, acceptability of treatment, adherence to homework, therapist adherence, and feasibility of assessments all ranked as “excellent” based on the a priori benchmarks [4]. Feasibility of recruitment was characterized as “good” given that 5 (71%) out of 7 of those approached enrolled in treatment.

### Quantitative Outcomes

Descriptive statistics for each intervention target (ie, pain catastrophizing, mindfulness, fear avoidance behaviors, limiting behaviors, and all-or-nothing behaviors) and outcome (ie, postconcussive symptoms, physical function, anxiety, depression, pain at rest, and pain during activity) are displayed in Table 4 with results of the 2-tailed paired *t* tests. Other measures outside of the study model (ie, all-or-nothing behaviors and depression) are also included.

**Table 4:** Change in study measures^a^.

Measure	T1, mean (SD)	T2, mean (SD)	T3, mean (SD)	Mean change (T1-T2)	Mean change (T1-T3)	*t* values (T1-T2), 95% (CI)	*t* values (T1-T3), 95% (CI)	Cohen *d* (T1-T2)	Cohen *d* (T1-T3)
Concussion symptoms (PCSS^b^)	68.80 (38.09)	24.60 (15.57)	24.40 (16.98)	44.20	44.40	2.91 (2.01-86.39)	3.65 (10.66-78.14)	1.30^c^	1.63^c^
Pain at rest (NRS^d^)	4.40 (3.05)	1.60 (1.52)	2.20 (2.95)	2.80	2.20	2.12 (–0.86 to 6.46)	2.75 (–0.021 to 4.42)	0.95^e^	1.23^c^
Pain during activity (NRS)	5.50 (2.86)	3.00 (1.22)	3.00 (4.12)	2.80	2.80	2.33 (–0.5 to 6.13)	2.89 (0.12-5.49)	1.04^e^	1.29^c^
Physical function (WHODAS^f^)	14.60 (9.84)	9.60 (5.03)	4.60 (5.41)	5.00	10.00	0.92 (–10.03 to 20.03)	1.59 (–7.45 to 27.45)	0.41^g^	0.71^h^
Pain catastrophizing (PCS^i^)	15.20 (7.53)	2.60 (2.19)	8.40 (9.04)	9.80	4.00	4.20 (3.32-16.28)	0.85 (–9.02 to 17.02)	1.88^c^	0.38^g^
Anxiety (GAD-7^j^)	14.60 (5.27)	6.40 (4.04)	7.60 (7.64)	8.20	7.00	2.59 (–0.60 to 17.00)	4.11 (2.27-11.73)	1.16^e^	1.05^e^
Anxiety (HADS-A^k^)	11.00 (3.46)	6.40 (3.14)	5.60 (3.51)	4.60	5.40	2.13 (–1.39 to 10.59)	3.52 (1.13-9.67)	0.95^e^	1.58^c^
Depression (HADS-D^l^)	7.20 (2.17)	2.20 (1.30)	3.80 (4.60)	5.00	3.40	3.54 (1.07-8.93)	3.03 (0.28-6.52)	1.58^c^	1.36^c^
Limiting (BRIQ^m^)	16.2 (3.96)	7.60 (3.85)	3.60 (3.78)	8.60	12.60	4.92 (3.74-13.46)	4.14 (5.15-21.05)	2.20^c^	1.85^c^
All or nothing (BRIQ)	13.20 (8.44)	7.00 (3.16)	9.20 (4.27)	6.20	4.00	1.60 (–4.57 to 16.97)	0.89 (–8.51 to 16.51)	0.72^h^	0.40^g^
Fear avoidance (FAB-TBI^n^)	47.40 (7.33)	24.60 (7.12)	30.00 (10.89)	22.80	17.40	3.88 (6.48-39.12)	6.13 (9.52-25.28)	1.74^c^	2.74^c^
Mindfulness (CAMS-R^o^)	26.20 (6.22)	32.40 (.55)	33.00 (1.87)	–6.20	–6.80	–2.05 (–14.59 to 2.19)	2.47 (–16.07 to 2.47)	–0.92^e^	–0.91^e^

^e^Effect sizes and scores on study measures before, after, and 3 months after completion of the Toolkit for Optimal Recovery after Concussion program for young adults with a recent concussion and anxiety.

^b^PCSS: Post-Concussion Symptom Scale.

^c^Very large effect size.

^d^NRS: Numerical Rating Scale.

^e^Large effect size.

^f^WHODAS: World Health Organization Disability Assessment Scale.

^g^Small medium effect size.

^h^Medium large effect size.

^i^PCS: Pain Catastrophizing Scale*.*

^j^GAD-7: Generalized Anxiety Disorder-7.

^k^HADS-A: Hospital Anxiety and Depression Scale–Anxiety.

^l^HADS-D: Hospital Anxiety and Depression Scale–Depression.

^m^BRIQ: Behavioral Response to Illness Questionnaire.

^n^FAB-TBI: Fear Avoidance Behavior after Traumatic Brain Injury.

^o^CAMS-R: Cognitive and Affective Mindfulness Scale-Revised

### Changes in Intervention Outcomes

Table 4 provides statistical details on changes in intervention outcomes. Briefly, changes between baseline and postintervention scores were characterized by a very large effect for postconcussive symptoms (*d*=1.30) and large effects for anxiety as measured by the GAD-7 (*d*=1.16) and HADS-A (*d*=0.95). Very large effects were consistent between preintervention and follow-up scores for postconcussive symptoms (*d*=1.63), and large effects were consistent for both GAD-7 (*d*=1.05) and HADS-A (*d*=1.57) measures for anxiety at follow-up. Physical function changed between baseline and immediate after the intervention with a small medium effect size (*d*=0.41), which grew at follow-up with a medium large effect size (*d*=0.71). Comparisons of pain at rest between baseline and postintervention scores yielded a large effect (*d*=0.95), which was retained as a very large effect at follow-up (*d*=1.23). Similarly, there was a large effect for improvement in pain during activity between baseline and immediately following intervention (*d*=1.04), which was maintained at follow-up (*d*=1.29). Analysis of depression scores between baseline and follow-up yielded a very large effect (*d*=1.58), which was maintained between baseline and follow-up time points (*d*=1.36).

### Changes in Intervention Targets

Table 4 provides statistical details on changes in intervention targets. Differences between baseline and postintervention scores yielded very large effects for improvements in pain catastrophizing (*d*=1.88), fear avoidance behavior (*d*=1.74), and limiting behaviors (*d*=2.20). These very large effects were maintained between baseline and follow-up scores for fear avoidance behaviors (*d*=2.74) and limiting behaviors (*d*=1.85). Large effects were observed from pre- to postintervention analyses of mindfulness scores (*d*=–0.92) and were maintained at follow-up (*d*=–0.91). Differences in pain catastrophizing scores between baseline and follow-up retained a small medium effect (*d*=0.38). Changes in all-or-nothing behaviors between baseline and postintervention time points held large effects (*d*=0.72), while change between baseline and follow-up time points was characterized by small medium effects (*d*=0.40).

### Qualitative Outcomes

RDA indicated impressions of high participant satisfaction with the program as well as perceptions of barriers and facilitators to adherence (Table 5). Overall, skills were viewed as generalizable and “eye-opening,” with 1 participant explaining that “I thought the strategies were very helpful not just for concussion symptoms, but also anxiety. I think it definitely had a positive impact.” Participants expressed appreciation toward mind-body skills for the induction of relaxation and the applicability of home practice despite initial skepticism. They also reported finding the acceptance techniques and the challenging unhelpful thought skills useful. One participant stated that “If it wasn’t for this program, I would never [be able to] back up from a thought and think about it and if it’s beneficial for me. It’s the reason I’m able to take a step back now.” Participants indicated that the focus on reengagement in activities helped reduce fear avoidance and indicated they would have liked more guidance around goal setting. They indicated that the concussion education content helped ameliorate their anxiety and that at times it conflicted with information they received from other providers, whose prescriptions focused on activity avoidance and limiting behaviors.

**Table 5:** Qualitative exit interviews^a^.

Program content			Impressions
**TOR-C^b^ skills and content**			
	General impressions	“It was really, really helpful and I think it could change my life forever.”	
	Mind-body skills	“I had a lot of anxiety and one of the tools that just worked was deep breathing.”	
	Engagement with activities	“At first, this was difficult because it was physically painful and also really discouraging. As the program went on, it was easier. It was good for me to push myself a little, and I actually went back to my activities.”	
	Information about concussion	“I had no idea concussion symptoms mirrored anxiety symptoms and that you can treat them in the same ways.”	
	CBT^c^ skills	“This program is helpful in that it is causing me to think about unhelpful thoughts instead of just experiencing them...I’m able to take a step back now and think about if it’s helpful for me.”	
	Home practice	“Before I go to bed, I like the body scan, the deep breathing—just trying to relax and not thinking so much because overthinking affects my sleep. Implementing the [mindfulness] strategies into my routine was really helpful.”	
**Barriers and facilitators for program engagement**			
	Barriers	“I didn’t do a very good job of implementing the tools that were given because I’m really busy, which I think a lot of others who are college students would be affected by.”	
	Facilitators	“The website, having that was helpful because...it was almost like a therapist to you but like outside of the session.”	

^a^Main themes arising from the postintervention qualitative exit interviews with 5 young adults with a recent concussion and anxiety who had completed the TOR-C program.

^b^TOR-C: Toolkit for Optimal Recovery after Concussion.

^c^CBT: cognitive behavioral therapy.

Participants indicated barriers as well as facilitators to program participation and adherence. Barriers included ambivalence about mindfulness, busyness, and symptom interference. One participant reported that they sometimes forgot to log their homework. Facilitators included early signals of improvement in symptoms, which increased motivation and was also bolstered by regular text reminders and use of the website, as well as trust in their clinician. Additional facilitators included a sense of responsibility to adhere to the program and the establishment of a routine for home practice. Overall, participants believed that the skills and lessons learned could be taken forward with one stating, “This program helps mentally finding ways I can use [skills] in everyday scenarios for the future.”

## Discussion

### Overview

Young adults with anxiety are prone to develop persistent concussion symptoms. We developed TOR-C, the first mind-body program aimed at preventing persistent concussion symptoms in this population. Here, we conducted a nonrandomized open pilot of TOR-C with exit interviews to test preliminary feasibility markers, signals of changes in outcomes, and perceptions of treatment, to refine the intervention in preparation for an upcoming pilot RCT.

### Principal Findings

TOR-C met or exceeded a priori feasibility benchmarks. Client satisfaction score, credibility, expectancy, acceptability of treatment, and adherence to home practice were excellent. Acceptability of treatment was excellent as well, with 100% of participants participating in 100% of sessions. Feasibility of recruitment was “good.” No adverse events related to the program were reported. Overall, these findings provide preliminary evidence that the TOR-C intervention and associated procedures are feasible and accepted by participants.

The TOR-C program was associated with a signal of improvement in intervention targets (ie, pain catastrophizing, mindfulness, fear avoidance, limiting behaviors, and all-or-nothing behaviors) and outcomes (anxiety, physical function, pain, depression, and postconcussion symptoms). The majority of effect sizes were large or very large. These findings show preliminary promise, though caution is warranted due to an inability to differentiate between the effects of the intervention and natural recovery in this trial.

Qualitative data from participants demonstrate satisfaction with the program as a tool for symptom relief, goal setting, concussion education, and maximizing accountability for skills use. Participant suggestions indicate a need for more experiential practice in session and in their daily lives. Other lessons learned include the importance of flexibility, more frequent check-ins about goals, and continuous encouragement for routine practice of skills. Participants with high symptom interference may benefit from a discussion reframing periods of increased discomfort as ideal for practicing skills. Overall, mind-body interventions for concussions may benefit from emphasizing the generalizability of mindfulness and the use of skills both in the presence and in the absence of symptoms.

### Limitations

Some limitations of this study should be noted. This was a nonrandomized open pilot with a small sample size, which is consistent with common procedures in the early stages of intervention development [40]. The primary aim was to establish preliminary feasibility of the program and test for signals of improvement in intervention targets and outcomes. In line with guidelines for feasibility trials [23], we also reported preliminary changes to intervention targets and outcomes. This study cannot yield reliable conclusions about the effects of TOR-C. Because participants experienced a recent concussion, many are expected to exhibit considerable improvement even in the absence of an intervention. Expectancy effects likely also contributed to improvement on the outcome measures. Findings should be interpreted as important preliminary data informing an upcoming feasibility RCT to reliably establish program feasibility against an active control intervention, consistent with the NCCIH framework for developing and testing mind-body interventions [23]. Future research should aim to replicate the findings as well as include a more diverse sample—specifically more Black, Indigenous, and other racial or ethnic minority individuals given the majority White sample—to provide generalizability of results and provide insight into care for underserved populations. Recruitment of a more diverse sample should also involve assessing for markers of socioeconomic status (eg, household income, insurance) to understand contextual factors that may impact participants’ concussion treatment and potential financial stressors that could exacerbate anxiety.

### Conclusions

TOR-C is the first mind-body program aiming to prevent persistent concussion symptoms among young adults with co-occurring anxiety. Findings suggest that TOR-C shows preliminary feasibility and has the potential to support recovery from concussion. We plan to leverage quantitative and qualitative findings to iteratively adapt the TOR-C in an upcoming feasibility RCT. Future iterations of TOR-C will include health education comparison conditions as well as larger sample sizes while incorporating participant feedback such as more frequent check-ins.

## Abbreviations

GAD-7: Generalized Anxiety Disorder-7
HADS: Hospital Anxiety and Depression Scale
HADS-A: Hospital Anxiety and Depression Scale–Anxiety
NCCIH: National Center for Complementary and Integrative Health
RCT: randomized controlled trial
REDCap: Research Electronic Data Capture
RDA: rapid data analysis
TOR-C: Toolkit for Optimal Recovery after Concussion

## Data Availability

The datasets generated during and/or analyzed during this study are available from the corresponding author on reasonable request.
